# Identification of an anatomical safe zone for humeral cerclage passage

**DOI:** 10.1016/j.jseint.2024.08.187

**Published:** 2024-08-30

**Authors:** Matthew T. Gulbrandsen, Lea E. McDaniel, Clayton H. Hui, Jeremy R. Brown, Taha M. Taka, Marc G. Lubitz, Anup A. Shah, Evan S. Lederman, Wesley P. Phipatanakul

**Affiliations:** a Department of Orthopedic Surgery, Loma Linda University School of Medicine, Loma Linda, CA, USA; b Department of Orthopedic Surgery, University of Arizona College of Medicine Phoenix, Phoenix, AZ, USA

**Keywords:** Humerus cerclage, Radial nerve anatomy, Shoulder arthroplasty, Proximal humerus fracture, Minimally invasive humerus cerclage, Radial nerve anatomic safe zone

## Abstract

**Background:**

Cerclage techniques have been used in the humerus in the setting of fractures and shoulder arthroplasty. Cerclage usage in the humerus has the potential to injure neurovascular structures. There is current literature describing deeper anatomic structures surrounding the humerus but not more superficial landmarks in reference to neurovascular structures. The purpose of this study was to determine safe zones for cerclage passage around the humerus.

**Methods:**

Eight fresh-frozen cadaveric specimens with no history of deformity, prior surgery, or trauma to the shoulder or arm were used in this study. A standard extended deltopectoral approach was performed in all 8 specimens. Dissection was performed to identify the various musculotendinous and neurovascular structures surrounding the humerus. Cerclage sutures were placed around the humerus. Measurements were made from the radial and axillary nerve to anatomic structures and the cerclage sutures.

**Results:**

The radial nerve entered the spiral groove on average 45.8 mm distal (range: 30.4 to 63.3 mm) to the inferior aspect of the pectoralis major tendon. Cerclage suture passed just distal to the inferior aspect of the pectoralis major tendon did not violate the radial nerve. The axillary nerve was located on the humerus an average of 5.3 mm (range: 2.4-10 mm) proximal to the superior aspect of the latissimus dorsi tendon insertion. A safe zone for cerclage passage was not identified distal to the radial nerve entering the spiral groove.

**Conclusion:**

The radial nerve entered the spiral groove on the humerus distal to the pectoralis insertion in all specimens. The axillary nerve started to contact the humerus proximal to the latissimus dorsi in all specimens. In this study, we found that cerclage passage medial to lateral from the latissimus dorsi proximally to the area just distal to the inferior pectoralis major insertion distally is a safe zone for cerclage passage.

The description of using cerclage to stabilize humerus fractures was first reported in French surgical literature in 1775.^6^ Cerclage techniques are commonly used in the humerus for fixation when treating periprosthetic humerus fractures, comminuted fractures, and revision shoulder arthroplasty.^1^^,^^2^ Cerclage fixation can provide an alternative to screw fixation during provisional fixation and definitive fixation in these complex cases secondary to fragment size and orientation and screw length limitations.

While an alternative to screw fixation, there are risks to using cerclage around the humerus due to the neurological anatomy surrounding the humeral shaft. There have been reports of radial nerve palsy in cases where cerclage wires were used in periprosthetic humerus fractures.^8^ Other studies note axillary nerve injury in humeral shaft fractures treated with cerclage wires.^3^ In addition to neurologic injuries, vascular and soft tissue injury can occur with improper cerclage wire placement.^10^ These injuries include incarceration of the brachial artery, deep brachial artery, and vascular supply to the periosteum.^10^ Given these potentially devastating complications, it is important to identify a safe zone using readily identifiable landmarks for cerclage passage as well as methodology for passing cerclage cables or suture.

In an effort to mitigate soft tissue envelope and blood supply compromise as well as a trend toward minimally invasive surgery, the identification of superficial anatomical landmarks to help create a safe zone for cerclage placement may avoid extensive dissection which potentially avoids the morbidity of neurovascular mobilization. The purpose of this study was to identify a safe zone for cerclage passage referencing reliable superficial anatomical structures. Our hypothesis is that there will be an anatomic safe zone in the humerus to safely pass cerclage based on superficial anatomic landmarks.

## Materials and methods

Eight fresh-frozen clavicle to finger-tip cadaveric specimens with no history of deformity, prior surgery, or trauma to the shoulder or arm were used in this study. Demographics included average age of 82.9 years (range: 74-99 years); 4 left and 4 right upper extremity specimens were dissected; 3 were male cadavers and 5 were female cadavers (Table I).

**Table I:** Demographics of donors.

Specimen #	Laterality	Sex	Age	Height (in)
1	L	F	83	66
2	L	M	81	67
3	R	F	87	67
4	R	M	84	67
5	R	F	99	60
6	L	F	74	69
7	R	F	74	69
8	L	M	81	71

*F*, female; *L*, left; *M*, male; *R*, right.

### Dissection approach

The cadavers were placed in supine position on a regular table. A standard extended deltopectoral approach was performed in all 8 specimens. An incision was made just lateral to the coracoid down along the deltoid toward the deltoid insertion then anterior to the lateral epicondyle. The deltopectoral interval was developed to identify the deltoid insertion. The exposure was continued lateral to the biceps and distally between the brachialis and brachioradialis interval. Measurements were performed in relation to identifiable anatomic structures to measure the distance to various neurovascular structures. Cerclage sutures (FiberTape; Arthrex Inc., Naples, FL, USA) were then passed medial to lateral with a cerclage passer, just distal to the distal margin of the pectoralis major tendon and proximal at the level of the latissimus tendon. In no cadavers was the radial nerve entrapped. The measurements were repeated in relation to the cerclage suture location. All measurements were made with the specimen in the supine position.

Measurements were performed using a digital caliper by a team including a sports medicine fellow and 2 residents. Any questions or disputes on measurements were answered by a separate sports medicine–trained orthopedic surgeon. A hemostat was clamped to the end of the neurovascular bundle proximal to the shoulder specimen to provide resting tension during measurements. Measurement uncertainty and descriptive statistics were derived for all measurements.

## Results

Measurements are summarized in Table II.

**Table II:** Measurements to various neurovascular structures (mm).

Structure	Mean	± 95% CI	Range	Min	Max
(1) Radial n. enters spiral groove distal to proximal deltoid insertion.	43	5.3	23.5	26.2	49.7
(2) Radial n. enters spiral groove to superior border of latissimus.	85.1	7.7	29.7	70.2	99.9
(3) Radial n. enters spiral groove and the inferior border of latissimus.	57.5	10.3	36.8	36.3	73.1
(4) Radial n. entrance into spiral groove and inferior border of the pectoralis major tendon.	45.8	8.2	32.9	30.4	63.3
(5) The upper border of the latissimus dorsi to the axillary nerve.	5.3	1.9	7.6	2.4	10
(6) The cerclage suture to the radial nerve entrance into spiral groove.	32.7	4.5	17.3	24.5	41.8
(7) The medial to lateral distance between humeral shaft to where the radial n. crosses at upper border of latissimus dorsi tendon.	15.2	4.9	20.9	6.1	27
(8) The medial to lateral distance between humeral shaft to where the radial n. crosses at the lower border of the latissimus dorsi tendon.	11.6	4	17.6	5.1	22.7
(9) Lateral epicondyle to radial n. exit from lateral spiral groove.	111.1	8.4	36.2	91.7	127.9
(10) Medial to lateral displacement of radial n. measured in elbow flexion.	61.3	10.1	36.3	42	78.3
(11) Medial to lateral displacement of radial n. in elbow extension.	38	11.6	46	16	62
(12) Radial nerve relative laxity or the change in excursion of the radial n. between elbow flexion and elbow extension.	23.3	7.7	34.5	13.2	47.7
(13) Medial to lateral distance from brachial artery and humerus distal to the radial n. exit from the spiral groove at its point of farthest displacement.	12.7	5.6	20.0	5.0	20.5

*n.*, nerve; *CI*, confidence interval; *Min*, minimum; *Max*, maximum.

### Key findings

**Figure 1:**
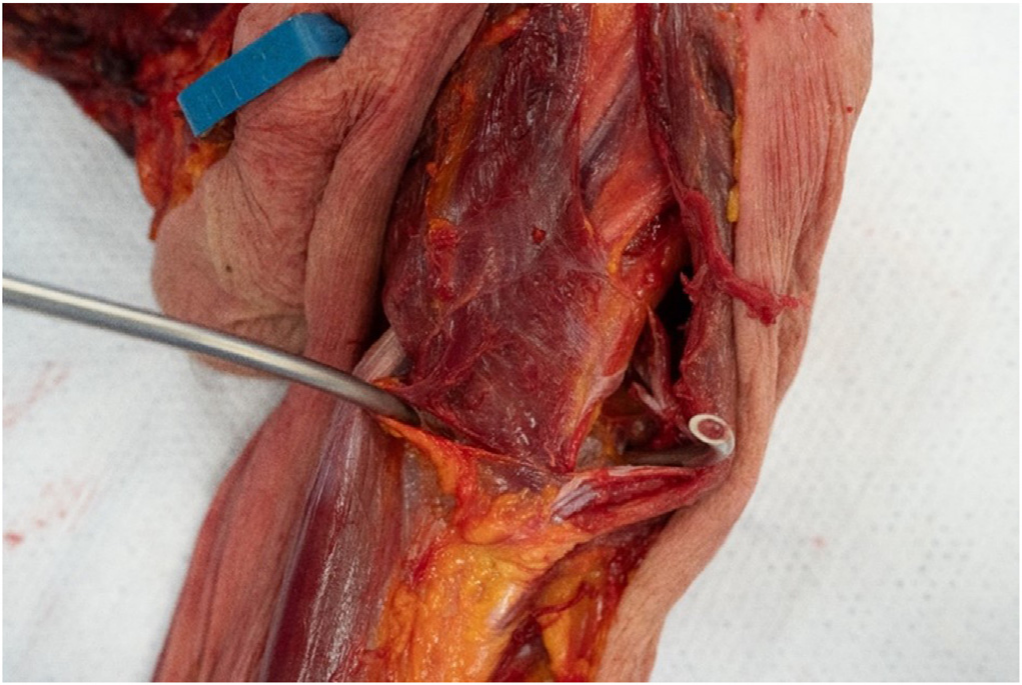
The cerclage passer just distal to the pectoralis major tendon (PT) in a superficial dissection of the humerus.

1. The radial nerve entered the spiral groove distal to the pectoralis insertion in all specimens. The shortest distance between the distal pectoralis tendon and radial nerve insertion into the spiral groove was 30.4 mm.
2. A cerclage suture was passed from medial to lateral just distal to the pectoralis major tendon and in no cadavers was the radial nerve entrapped. After passing the cerclage suture, the closest the cerclage measured to the radial nerve in the proximal spiral groove was 24.5 mm (Figure 1, Figure 2 and Figure 1, Figure 2).

**Figure 2:**
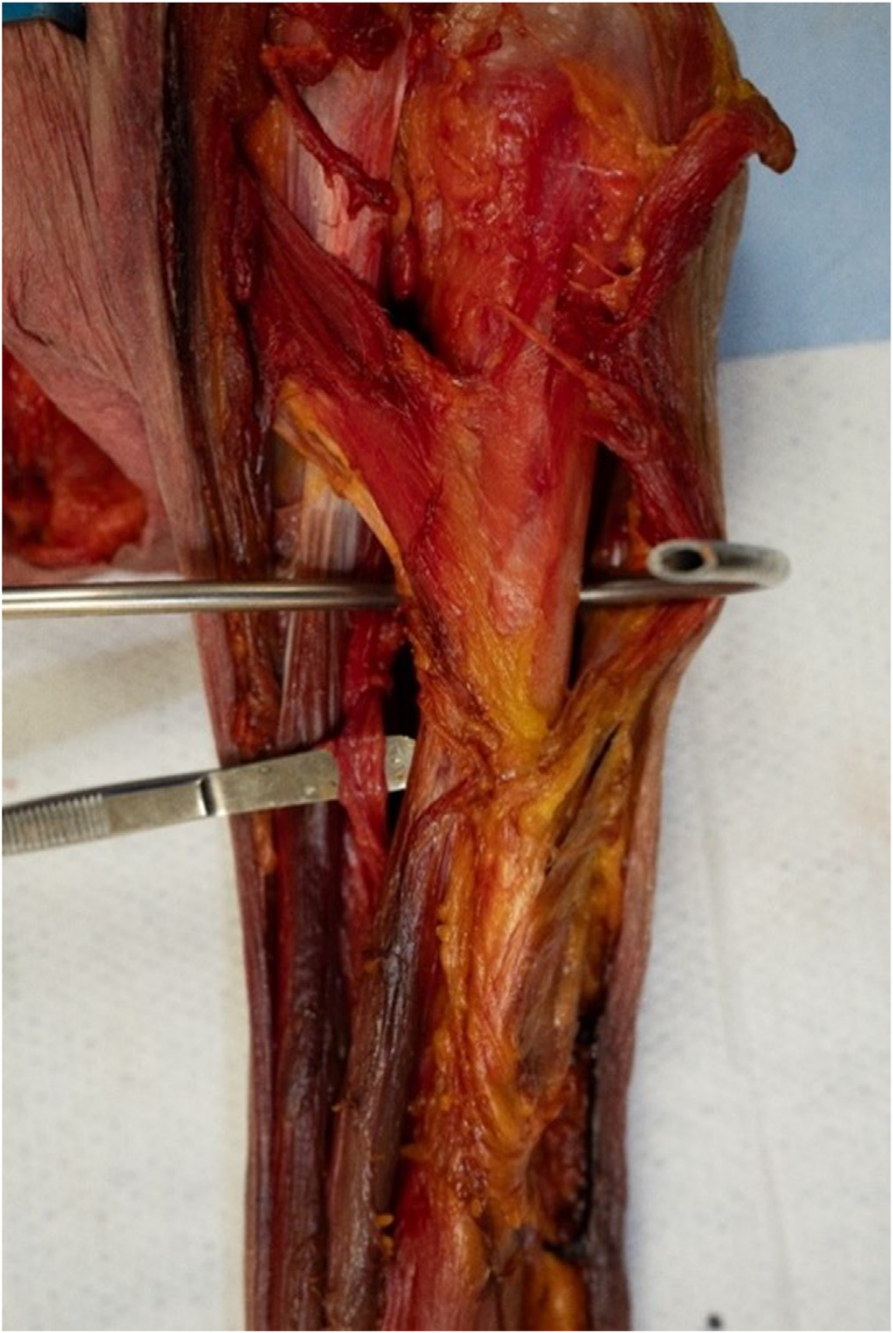
The cerclage passer placed from medial to lateral around the proximal humerus highlighting that it does not violate the radial nerve (R).
3. The mean distance between the upper border of latissimus dorsi and the axillary nerve proximally was 5.3 mm (range: 2.4-7.6 mm).
4. The mean medial to lateral distance between the humeral shaft and the radial nerve as it traverses the latissimus dorsi is between 15.2 (proximal latissimus tendon) and 11.6 mm (distal latissimus tendon).

The mean, standard deviation, standard median of error, measurement uncertainty, and minimum and maximum values were tabulated for all 8 of the specimens in the same technique described above (Table II).

## Discussion

**Figure 3:**
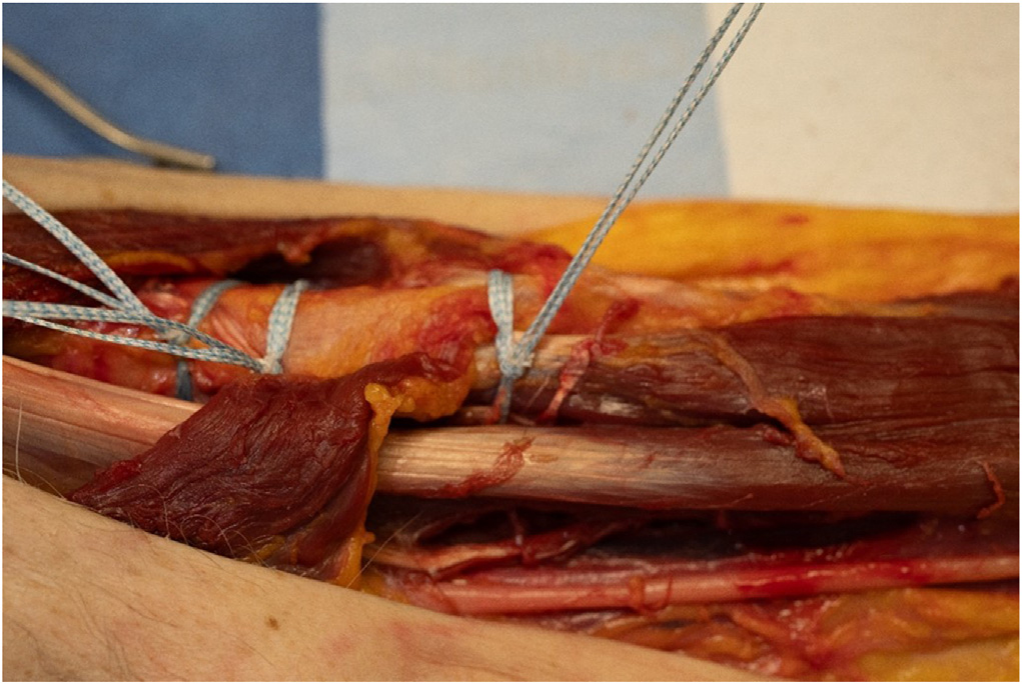
Cerclage sutures around the proximal humerus at various levels, 2 proximal to the pectoralis major insertion through the latissimus tendon, and 1 distal to the pectoralis major insertion. None of which violate the radial or axillary nerve.

**Figure 4:**
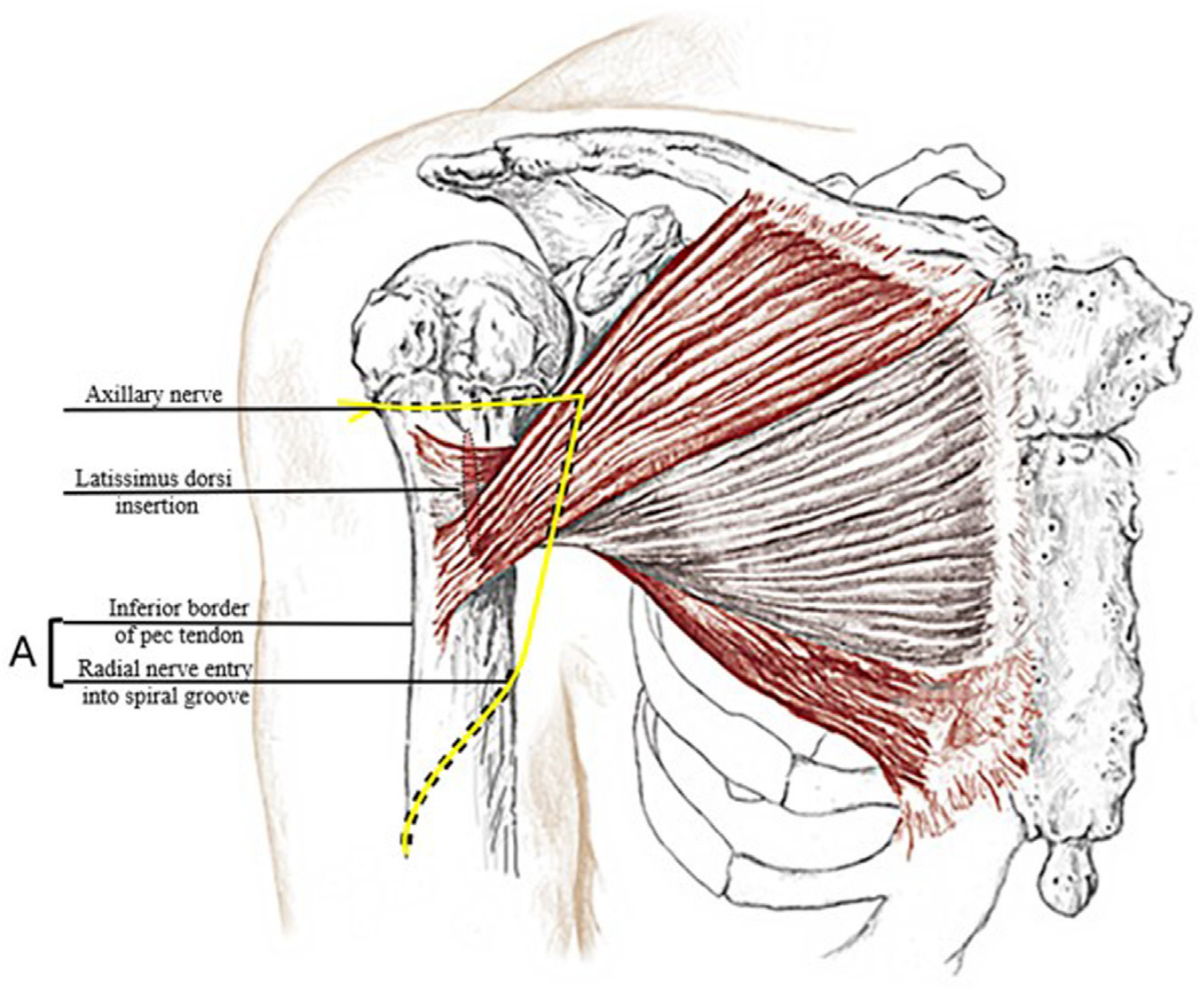
Drawing of pectoralis major insertion in relation to radial nerve. **(A)** The zone distal to the pectoralis insertion and proximal to the spiral groove. A *red* oval outlined in *black* represents the latissimus dorsi tendon insertion.

Cerclage cabling of the humerus can lead to potentially devastating neurovascular injury.^1^^,^^10^ Surgery techniques should aim to increase safety and decrease surgical morbidity. This study was conducted with the goal of determining an anatomic safe zone for cerclage placement technique from an anterior deltopectoral approach. Primarily, this study determined that there is a safe zone for cerclage passage in the proximal humerus using the superior border latissimus tendon proximally and the pectoralis major tendon insertion distally as anatomic landmarks for the axillary and radial nerves, respectively (Figure 3, Figure 4 and Figure 3, Figure 4). Additionally, passage of cerclage from a medial to lateral direction avoided neurovascular capture in all specimens.

Neurovascular proximity during placement of cerclage wire has been studied in the literature. Fu et al evaluated cerclage wire placement using the deltopectoral approach.^7^ Their study determined the average distance between the radial nerve entry into the spiral groove and the inferior margin of the latissimus was 39.6 mm, with one specimen showing it to be as close as 25.5 mm.^7^ The present study showed an increased distance of 57.5 mm between the radial nerve entry into the spiral groove and the inferior margin of the latissimus, with one specimen showing it to be as close as 36.3 mm. They did not report the relationship between the pectoralis and radial nerve and they released the pectoralis to expose the latissimus.^7^ The more superficial location of the pectoralis makes it a more accessible landmark compared to the inferior border of the latissimus which lies deeper and is not routinely exposed in a standard deltopectoral approach.

The approach for fixing humerus fractures does not typically require a pectoralis release to expose the inferior latissimus which is a more medial and deeper structure. At times, the proximal fracture fragment of the humerus is abducted by the force of the deltoid,^4^ and the inferior pectoralis serves as a readily identifiable, more superficial landmark for cerclage reduction techniques, which could be performed through a more minimally invasive technique when desired.

Gipsman et al also studied cerclage passage in the proximal humerus. They divided the proximal humerus into 4 zones.^9^ Zone 1 was from proximal pectoralis major insertion to proximal deltoid insertion, zone 2 was from proximal deltoid insertion to distal pectoralis major tendon insertion, zone 3 was from distal pectoralis major insertion to distal deltoid insertion, and zone 4 was the area distal to the distal deltoid insertion.^9^ Interestingly, they reported that 50% of their specimen showed the radial nerve to enter the spiral groove proximal to the distal margin of the pectoralis major insertion which was not the case in our study.^9^ We found that in all specimens, the radial nerve entered the spiral groove distal to the distal margin of the pectoralis major insertion.

Kadar et al passed cerclage around the humerus superficially without any dissection at a distance of 30%, 45%, and 60% relative to the height of the humerus from proximal to distal.^10^ They found that passing cerclage at 30% and 60% distal to the proximal tip of the humerus resulted in no damage to the radial nerve or brachial artery,^10^ but passing suture at 45% caused damage to neurovascular structures.^10^ Although this technique may be considered safe, it may be less reliable with distorted anatomy in fractures and arthroplasty applications.

In the present study, we passed suture cerclage medial to lateral, just inferior to the pectoralis tendon insertion. Considering that the radial nerve lies a mean distance of 15.2 mm and 11.6 mm medial to the humerus at superior and inferior latissimus tendon, respectively, lateral to medial cerclage passage, without direct palpation or visualization of the cerclage passer on medial humerus cortex, could lead to a higher risk of radial nerve entrapment. The authors recommend placing the passers from medial to lateral proximal to the spiral groove since they can be controlled more precisely at the point of entry reducing the risk of injury. In all specimens, the suture cerclage did not contact or entrap the radial nerve. Specifically, we found that the radial nerve entered the spiral groove on average 45.8 mm distal to the inferior pectoralis major tendon insertion and that the closest distance from radial nerve to inferior margin of pectoralis major was 30.4 mm. In this study, the zone located just distal to the pectoralis insertion emerged as a reliable and safe area for conducting cerclage procedures without the need to dissect deeper to identify the distal latissimus dorsi insertion.

**Figure 5:**
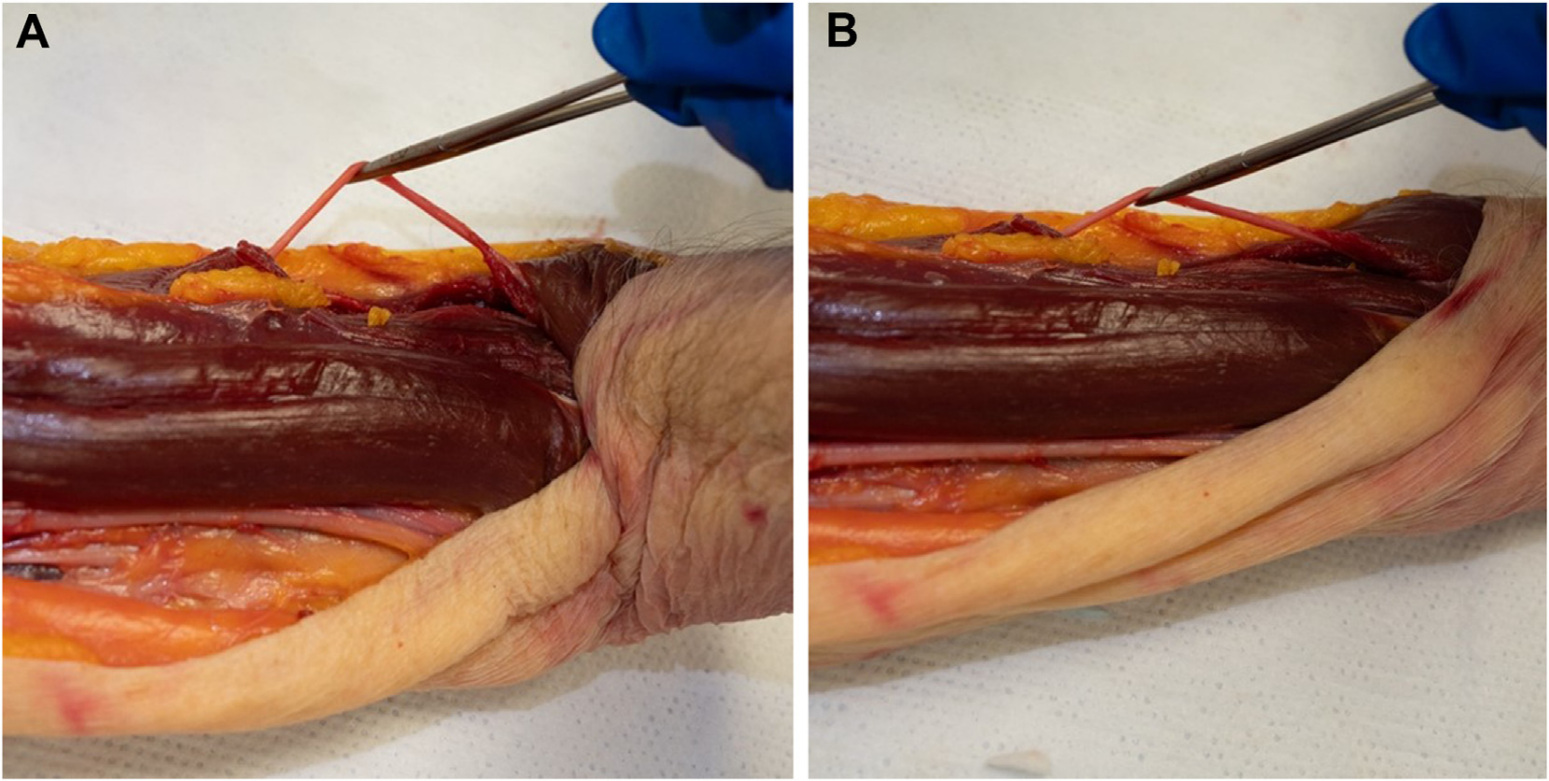
Images of the radial nerve distal to its exit from the radial groove at the point of maximal distance from the humerus. (**A**) The distance during elbow flexion. **(B**) The distance during elbow extension.

A previous cadaveric study by Chen et al investigated how elbow flexion can affect the excursion of the radial nerve.^5^ Chen et al determined that flexing the elbow and releasing the lateral intermuscular septum of the brachium increased the distance between the humerus shaft and that radial nerve.^5^ The present study found that when the elbow was extended, at the point of farthest excursion, the radial nerve was on average 38.0 mm from the medial humerus shaft after dissection (Fig. 5, *B*), with flexion to 90° this distance increased to an average of 61.3 mm (Fig. 5, *A*). This suggests that on average, the radial nerve moved 23.3 mm medially away from the medial humerus with elbow flexion. More excursion of the radial nerve was possible if the elbow was flexed allowing greater mobility and possibly reduced risk of injury during mobilization. This would suggest when placing distal cerclage that the safer position is elbow flexion but requires exposure of the nerve for safety.

Distally, one must also take into account the brachial artery. In our study, the brachial artery was always within 20.5 mm of the humerus at all points. In one specimen, the farthest distance the brachial artery ever reached was 5.0 mm. Given these findings in regarding the brachial artery and the radial nerve, we did not identify a safe zone for distal humerus suture passage without dissection that exposed these neurovascular structures. This small distance is also a reason to pass cerclage medial to lateral to be able to directly visualize the avoidance of the brachial artery.

When using cerclage more proximally, the axillary nerve is the structure most at risk. Previous studies have shown a relative safe zone in reference to the deltoid insertion. In a previous study, Mekhail et al determined the distance between the axillary nerve and the proximal border of the anterior deltoid tendon to be 56 mm.^11^ Likewise, Moatshe et al indicated that the axillary nerve was positioned at least 36.1 mm proximal to the insertion point of the deltoid tendon.^12^ In the present study, we focused on the distance between the axillary nerve and superior edge of the latissimus which is readily visualized during standard deltopectoral approaches. We measured the distance from the superior border of the latissimus to the axillary nerve to be on average 5.3 mm (range: 2.4-10.0 mm). This suggests that cerclage passage proximal to the proximal margin of the latissimus tendon is more precarious and one should consider visualizing, palpating, and protecting the axillary nerve when using cerclage proximal to the proximal edge of the latissimus dorsi tendon. The nerve can be easily identified and does not require releases or extensive dissection. Passage of the suture through the latissimus inferior to the superior border was considered the upper border of the safe zone.

This study has several important limitations. First, our study examined the anatomic relationship with an intact humerus structure in the absence of fractures or scarring that might otherwise distort the positioning of anatomic structures. Additionally, owing to the nature of cadaveric studies, the muscle tissue encountered in our study had undergone postmortem changes which may alter the shape and tension of the encountered landmarks and subsequently the anatomic relation of the adjacent neurovascular bundles. These were also not full cadavers but rather clavicle to fingertip cadaver specimen, which could have affected tension. Although we attempted to combat this by tensioning the neurovascular structures at the proximal portion of the cadaver, some variance is likely to still occur. Finally, our study documents the age, gender, and height of specimens used; however, we did not consider anatomic variability based on ethnicity of the specimens.

## Conclusion

The radial nerve entered the spiral groove on the humerus distal to the pectoralis insertion in all specimens. The axillary nerve started to contact the humerus proximal to the latissimus dorsi in all specimens. In this study, we found that cerclage passage medial to lateral from the latissimus dorsi proximally to the area just distal to the inferior pectoralis major insertion distally is safe and is a safe zone for cerclage passage.

## Disclaimers:

Funding: This study was funded by a grant from 10.13039/100007307Arthrex Inc. Cadavers and Fibertape cerclage sutures were provided by Arthrex Inc.

Conflicts of interest: Dr. Wesley P. Phipatanakul is a consultant for Arthrex. Dr. Evan Lederman is a consultant for Arthrex; received research support from Arthrex; received royalties from Arthrex. Dr. Anup Shah is a consultant for Arthrex; received research support from Arthrex; received royalties from Medacta. The other authors, their immediate families, and any research foundation with which they are affiliated have not received any financial payments or other benefits from any commercial entity related to the subject of this article.
